# Time dependency in the radiofrequency lesion formation for a local impedance guided catheter in an ex vivo experimental model

**DOI:** 10.1002/joa3.12789

**Published:** 2022-10-13

**Authors:** Daisuke Kawano, Hitoshi Mori, Kenta Tsutsui, Hidehira Fukaya, Naomichi Tanaka, Masataka Narita, Wataru Sasaki, Kazuhisa Matsumoto, Yoshifumi Ikeda, Takahide Arai, Shintaro Nakano, Ritsushi Kato

**Affiliations:** ^1^ Department of Cardiology Saitama Medical University, International Medical Center Hidaka Japan; ^2^ Department of Cardiovascular Medicine Kitasato University School of Medicine Sagamihara Japan

**Keywords:** contact force, in‐vitro experiment, lesion size, local impedance, radiofrequency ablation

## Abstract

**Background:**

The local impedance (LI) is an emerging technology that monitors tissue‐catheter coupling during radiofrequency (RF) ablation. The relationships between the LI, RF delivery time, and lesion formation remain unclear.

**Methods:**

Using an LI‐enabled RF catheter in an ex vivo experimental model, RF lesions were created combined with various steps in the power (40 and 50 W), CF (10 g, 30 g, and 50 g), and time (10s, 20s, 30s, 40s, 50s, and 60s at 40 W and 5 s, 10s, 20s, 30s, 40s, 50s, and 60s at 50 W). The correlations between the LI drop, lesion size, and RF delivery time were evaluated. The rate of change in the time‐dependent gain in the LI, depth, and diameter and the time to reach 90% decay of the peak dY/dT (time to 90% decay) were assessed.

**Results:**

The correlation between the LI drop and ablation time revealed non‐linear changes. The time to a 90% decay in the LI drop differed depending on the RF ablation setting and was always shorter with the 50 W setting than 40 W setting. The LI drop always correlated with the lesion formation under all ablation power settings. Deeper or wider lesions were predominantly created within the time to 90% decay of the LI drop.

**Conclusion:**

The LI drop was useful for predicting lesion sizes. Deeper or wider lesions cannot be obtained with a longer ablation than the 90% decay time of the LI drop. A shorter ablation than the 90% decay time of the LI drop would be preferable for an effective ablation.

## 1 BACKGROUND

Radiofrequency (RF) catheter ablation is an effective treatment of cardiac arrhythmias in which a “durable” transmural RF lesion formation is required.^1^ It has been demonstrated that myriads of factors, e.g., the delivery time,^2^, ^3^ contact force (CF),^4^, ^5^ irrigation flow rate,^6^, ^7^, ^8^ catheter contact angle,^9^ catheter contact area,^10^ tissue property, blood flow, etc. play roles in the lesion formation. Several attempts have been made to establish a predictive model of the lesion formation. Composite component scoring systems such as the “ablation index” (AI, Biosense Webster) or “lesion size index” (LSI, Abbott) incorporate catheter‐sided parameters (CF, time, power, and electrical current in a weighted formula).^11^, ^12^ Those indices increase in a time‐dependent manner and are capable of a lesion size prediction to some extent. Such an “index‐based” ablation is associated with improved clinical outcomes.^13^, ^14^ However, those parameters do not reflect the tissue characteristics and cannot predict steam pops.^15^

More recently, a newer technology that monitors the “local impedance (LI)” around the catheter‐tissue interface has been introduced (DIRECTSENSE, Boston Scientific, Maple Grove, MN).^16^, ^17^ The LI increases in response to an increased CF before ablation and drops when the tissue is heated during RF ablation. Cross‐sectional analyses of 60‐s lesions indicate that the degree of the “LI drop” correlates with the lesion formation.^18^, ^19^ However, the lack of any longitudinal data hampers a direct translation of the LI drop's ability to predict the lesion size for clinical use. An excessively longer ablation time cannot necessarily create a greater ablation lesion and has a risk of steam pops or collateral tissue damage. We aimed to investigate the association between the RF delivery time and lesion formation using the IntellaNav StablePoint™ catheter (Boston Scientific, Maple Grove, MN) in an ex vivo experimental model.

## 2 METHODS

### 2.1 Ex vivo experimental model

The experimental protocol was described previously.^19^ In short, a swine left ventricle slice was fixed on a plate in a bath filled with saline, which was circulated and heated by a peristatic pump with a thermostat. The pool temperature was kept at 37°C. An IntellaNav StablePoint™ catheter was stabilized manually through a plastic pipe and placed perpendicular to the tissue. The irrigation flow rate was 30 ml/min during RF applications.

### 2.2 RF energy delivery protocol

The experiment was performed with a Rhythmia™ Mapping System (Boston Scientific, Maple Grove, MN). In our previous paper, we found that a 30 W ablation is underpowered due to the tip length,^19^ however, the maximum ablation power of the StablePoint™ catheter is 50 W. Therefore, the ablation in this study was performed with 40 W or 50 W. RF energy was delivered with a CF of 10 g, 30 g, and 50 g and various times (40 W:10s, 20s, 30s, 40s, 50s, and 60s; and 50W:5s, 10s, 20s, 30s, 40s, 50s, and 60s). Steam pops were defined as audible pops. Even when a steam pop occurred, the RF energy delivery was continued. The experiment was repeated 10 times at each setting and all data were analyzed.

### 2.3 Local impedance measurement

The LI was measured between the entire distal tip (4 mm tip) and 2nd ring electrode. The rate of change in the time dependent gain in the measured LI was assessed, and the time to reach a 90% decay in the peak dY/dt, where Y indicated the LI value, was expressed as the “time to a 90% decay”. The LI drop, which correlates with the local tissue temperature,^16^ rapidly increased before reaching the “time to a 90% decay” (rapidly increasing phase), while it slowly increased after reaching this point (slowly increasing phase).

### 2.4 RF lesion assessment

The lesion border was defined as a change in the tissue color. As with our previous experiment, the maximum depth (a), maximum diameter (b), depth at the maximum diameter (c), and surface maximum diameter (d) of the lesion were measured.^19^ Then, the lesion volume was calculated as: volume = (1/6) × π × (a × b^2^ + c × d^2^/2). The rate of change in the time dependent gain in the measured lesion size was also assessed, and the time to reach a 90% decay in the peak dY/dt, where Y indicated the LI drop, lesion depth or lesion diameter, was expressed as the “time to a 90% decay”.

### 2.5 Statistical analysis

The statistical analyses were performed using MP® Pro software, version 16.0 (SAS Institute) and GraphPad Prism9 software (GraphPad Software inc, San Diego, CA). The Cochran‐Armitage test was used for the trend analyses. A correlation analysis was performed using a Pearson's correlation analysis (*r*). A non‐linear regression analysis was performed to investigate the relationship between the RF delivery time, LI drop, and lesion formation (lesion depth, lesion diameter, and lesion volume). A value of *p* < .05 was considered statistically significance, unless specified otherwise.

## 3 RESULTS

### 3.1 Relationship between the LI drop and RF delivery time

**FIGURE 1:**
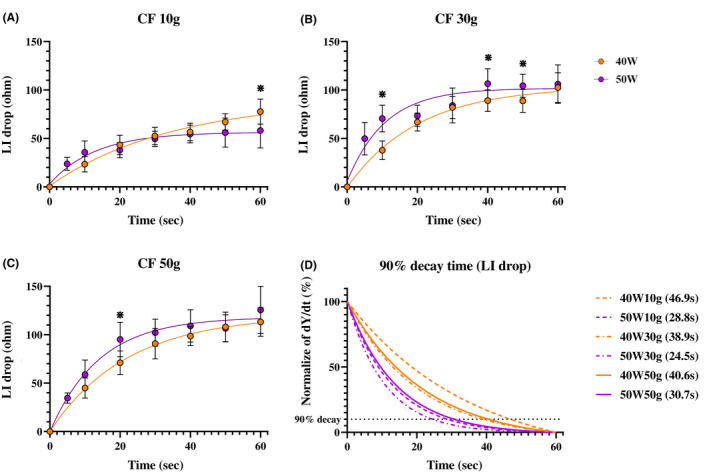
(A–C) correlation between the LI drop and RF delivery time. A non‐linear fit was performed by prism. A statistically significant difference between the 40 W and 50 W power settings is shown by the asterisk (*). A time‐dependent increase in the LI drop was observed with all power and CF settings. (D) Analysis of the time to reach 90%. The rate of change in the time‐dependent change in the measured LI was assessed, and the time to reach a 90% decay in the peak dY/dT, where Y indicates the LI value, was expressed as the ‘time to a 90% decay”. The phase before reaching the “time to a 90% decay” was always shorter with the 50 W ablation than 40 W ablation.

**FIGURE 2:**
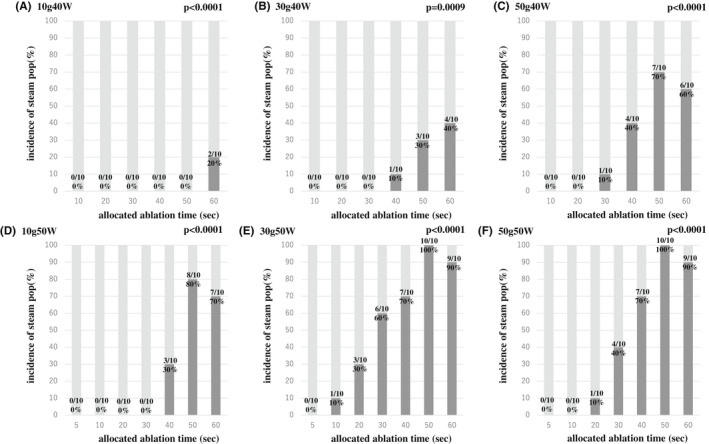
The incidence of steam pops under each ablation setting. The longer ablation deliveries had a higher incidence of steam pops (Cochran‐Armitage analysis). (A–C) Ablation power with 40 W, (D–F) Ablation power with 50 W.

Figure 1A–C shows the relationship between the LI drop and RF delivery time. A non‐linear, time‐dependent increase in the LI drop was observed for all power and CF settings (CF 10 g, Figure 1A, CF 30 g, Figure 1B, CF 50 g, and Figure 1C, power is color‐coded in each panel). The rapidly increasing phase, which was defined as the phase before reaching the “time to a 90% decay”, was always longer at a 40 W ablation than 50 W ablation (Figure 1D and Table 1, Time to a 90% decay in the LI drop; 40 W10 g, 46.9 s; 40 W30 g, 38.9 s; 40 W50 g, 40.6 s; 50 W10 g, 28.8 s; 50 W30 g, 24.5 s; and 50 W50 g, 30.7 s). Figure 2A–F shows the incidence of steam pops under each ablation setting. The longer ablation deliveries had a higher incidence of steam pops.

**TABLE 1:** The 90% decay time of each parameter

		LI drop	Lesion depth	Lesion diameter
**Ablation power: 40 W**				
CF	10 g	46.9 s	37.0 s	26.2 s
	30 g	38.9 s	37.0 s	22.2 s
	50 g	40.6 s	33.3 s	21.0 s
**Ablation power: 50 W**				
CF	10 g	28.8 s	28.3 s	23.7 s
	30 g	24.5 s	12.4 s	11.9 s
	50 g	30.8 s	22.5 s	22.2 s

### 3.2 Relationship between the RF delivery time and lesion formation

**FIGURE 3:**
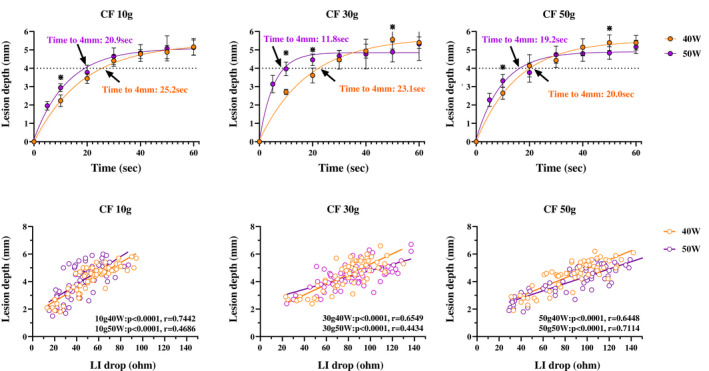
(A) Relationship between the RF delivery time and lesion depth. A non‐linear fit was performed by prism. A non‐linear, time‐dependent increase in the lesion depth was observed with all power and CF settings. The RF delivery time required to reach a 4 mm lesion depth was as follows: 40 W10 g: 25.9 s, 40 W30 g: 23.1 s, 40 W50 g: 20.0 s, 50 W10 g: 20.9 s, 50 W30 g: 11.8 s, and 50 W50 g; 19.2 s. (B) Relationship between the LI drop and lesion depth. A linear and positive correlation was observed with all ablation settings.

Figure 3A shows the relationship between the RF delivery time and lesion depth. A non‐linear, time‐dependent increase in the lesion depth was observed for all power and CF settings (40 W10 g; *R* ^2^ = 0.9706, 40 W30 g; *R* ^2^ = 0.9549, 40 W50 g; *R* ^2^ = 0.9513, 50 W10 g; *R* ^2^ = 0.9440, 50 W30 g; *R* ^2^ = 0.8908, and 50 W50 g; *R* ^2^ = 0.9336). The RF delivery time required to reach a 4 mm lesion depth was as follows: 40 W10 g: 25.9 s, 40 W30 g: 23.1 s, 40 W50 g: 20.0 s, 50 W10 g: 20.9 s, 50 W30 g: 11.8 s, and 50 W50 g; 19.2 s.

The time to a 90% decay for the lesion depth was as follows: 40 W10 g: 37.0 s, 40 W30 g: 37.0 s, 40 W50 g: 33.3 s, 50 W10 g: 28.3 s, 50 W30 g: 12.4 s, and 50 W50 g: 22.5 s (Table 1). That was shorter than that of the LI drop under each setting. Figure 3B shows the relationship between the LI drop and lesion depth. A linear and positive correlation was observed for all ablation settings.

**FIGURE 4:**
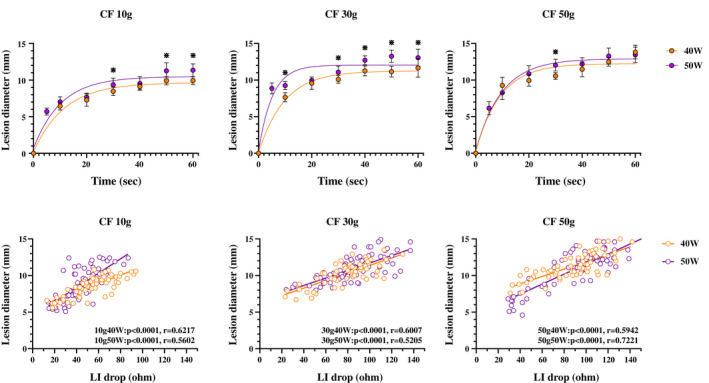
(A) Relationship between the RF delivery time and lesion diameter. A statistically significant difference between the 40 W and 50 W power settings is shown by the asterisk (*). A non‐linear fit was performed by prism. A non‐linear and time‐dependent increase in the lesion diameter was also observed with all power and CF settings. (B) the relationship between the LI drop and lesion diameter. A linear and positive correlation was observed with all ablation settings.

Figure 4A shows the relationship between the RF delivery time and lesion diameter. A non‐linear, time‐dependent increase in the lesion diameter was also observed for all power and CF settings (40 W10 g; *R* ^2^ = 0.9522, 40 W30 g; *R* ^2^ = 0.9567, 40 W50 g; *R* ^2^ = 0.9179, 50 W10 g; *R* ^2^ = 0.8921, 50 W30 g; *R* ^2^ = 0.8960, and 50 W50 g; *R* ^2^ = 0.9498). The time to a 90% decay for the lesion diameter was as follows: 40 W10 g: 26.2 s, 40 W30 g: 22.2 s, 40 W50 g: 21.0 s, 50 W10 g: 23.7 s, 50 W30 g: 11.9 s, and 50 W50 g; 22.2 s (Table 1). That was also shorter than that of the LI drop under each setting. Figure 4B shows the relationship between the LI drop and lesion diameter. A linear and positive correlation was observed for all ablation settings.

**FIGURE 5:**
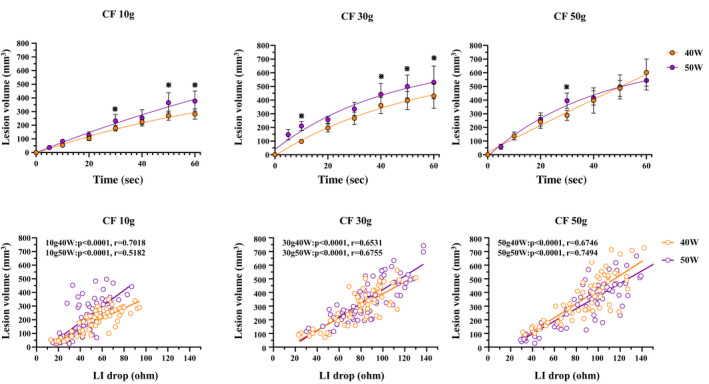
(A) Relationship between the RF delivery time and lesion volume. The time groups with a statistically significant difference between the 40 W and 50 W power settings are marked by an asterisk (*). A non‐linear fit was performed by prism. A positive correlation between the RF delivery time and lesion volume was observed under all power settings. (B) the relationship between the LI drop and lesion volume. A linear and positive correlation was also observed with all ablation settings.

Figure 5A shows the relationship between the RF delivery time and lesion volume. A linear, positive correlation between the RF delivery time and lesion volume was observed for all power settings (40 W10 g; *R* ^2^ = 0.9462, 40 W30 g; *R* ^2^ = 0.8952, 40 W50 g; *R* ^2^ = 0.9073, 50 W10 g; *R* ^2^ = 0.8880, 50 W30 g; *R* ^2^ = 0.8654, and 50 W50 g; *R* ^2^ = 0.9314). Figure 5B shows the relationship between the LI drop and lesion volume. A linear and positive correlation was also observed for all ablation settings.

## 4 DISCUSSION

### 4.1 Major findings

The major findings of our study were as follows:

1. The LI drop revealed a non‐linear change, and the rapidly increasing phases differed depending on the RF ablation setting and were shorter for the 50 W group.
2. The LI drop always correlated with the lesion depth, lesion diameter, and lesion volume under all ablation power settings.
3. Deep and wide lesions were predominantly created during the rapidly increasing phase of the LI drop.

### 4.2 Relationship between the LI drop and RF delivery time

The LI is correlated with the local tissue temperature.^16^ In this study, we analyzed the time course of the LI drop by dividing it into two phases: rapidly increasing phase and slowly increasing phase. During the RF ablation, the ablation lesions were created by two tissue heating mechanisms: resistive heating (rapid and active) and conductive heating (slow and passive).^20^ The lesion formation during the rapidly increasing phase of the LI drop, which suggested that the local tissue temperature was rapidly increasing, was mainly created with rapid and active heating, while that during the slowly increasing phase of the LI drop, which suggested that the local tissue temperature reached a plateau level, was mainly created with slow and passive heating. The 90% decay time of the LI drop differed between 40 W and 50 W and was shorter with the 50 W setting, which suggested that the ablation lesion was created within a shorter ablation time when using rapid and active heating under the 50 W ablation. After reaching the slowly increasing phase, the ablation lesion slowly created the lesion with slow and passive heating.

### 4.3 Relationship between the lesion formation and LI drop

Our previous study reported that there is a positive correlation between the LI drop and lesion volume and diameter, while the lesion depth does not follow an increase in the LI drop.^19^ However, our present study demonstrated that the LI drop correlated with the lesion depth, lesion diameter, and lesion volume, respectively. The ablation time in our previous study was 60 s for a quantitative purpose to compare the lesion characteristics of the StablePoint™ catheter with other ablation catheters. This study demonstrated that the 90% decay time of the lesion depth was shorter than that of the LI drop, and the LI drop slightly kept on increasing after reaching the 90% decay time. Under the 60‐s ablation in our previous study, the LI drop increased even though the lesion depth had already reached the 90% decay time. That discrepancy resulted in our previous result of no correlation between the lesion depth and LI drop. Our present study suggested that the LI drop was an accurate indicator of the lesion formation before reaching the 90% decay time. Furthermore, the time to a 90% decay of the lesion depth or lesion diameter was shorter than that of the LI drop under each setting (Table 1). That indicated that a deep lesion or wide lesion was created during the rapidly increasing phase of the LI drop. In contrast to the non‐linear correlation between the depth or diameter and the ablation time, that of the lesion volume and ablation time revealed a linear correlation. In this study, the ablation time was set at less than 60 s. When the ablation time was set for longer than 60 s, the correlation between the lesion volume and ablation time showed a non‐linear correlation.

Our previous study reported that steam pops occurred more frequently at a high wattage ablation.^19^ In this study, the rapidly increasing phase in the 50 W group was always shorter than that in the 40 W group. This suggested that the tissue temperature with the 50 W ablation was rapidly rising more than that with the 40 W ablation. As a result, steam pops occurred more frequently with the 50 W ablation even though the degree of the LI drop was similar. Our present study showed that the longer ablation deliveries had a higher incidence of steam pops (Figure 2). However, steam pop occurred within a short ablation time in 50 W settings (Figure 2E,F). This would be caused by a rapid increase of local tissue temperature due to the rapid and active heating. We previously revealed that LI drop predicted a steam pop with a cutoff value of 89 Ω. Monitoring of LI drop is important to prevent the steam pop in high power ablation.

### 4.4 The influence of the tip length on the lesion formation

It has been reported that the lesion formation becomes larger with time when using a conventional 3.5 mm tip length catheter.^21^ However, the lesion depth and diameter created with the StablePoint™ catheter reached the 90% decay around 40 s. The tip length of the Stable Point™ is 4.0 mm. The surface area of the tip of the Stable Point™ is appropriately 15% larger than that of the 3.5 mm tip catheter.^22^ Therefore, with a larger catheter tip, a larger part is exposed to the blood pool and more RF energy is dissipated into the blood pool. As a result, it might be difficult to reach a greater lesion depth or diameter with a longer RF energy delivery.

### 4.5 Optimal RF delivery time during pulmonary vein isolation of atrial fibrillation

Pulmonary vein isolation (PVI) is a cornerstone therapeutic strategy for the treatment of atrial fibrillation. An excessively longer ablation time is not necessarily needed for the PVI, however, too short an RF is insufficient to create a durable PVI. Several studies have reported that the maximum wall thickness of the myocardial sleeves surrounding the pulmonary veins is less than 4 mm, with an average thickness of 2 mm.^23^, ^24^ Therefore, a lesion depth of >4 mm is generally considered essential for an intramural atrial lesion for atrial fibrillation ablation. We focused on (1) the RF delivery time to achieve the lesion depth (4 mm) and (2) the RF delivery time to reach a 90% decay time. Our findings suggested that an RF delivery time of 20.0–25.2 s under the 40 W setting and 11.8–20.9 s under the 50 W setting was needed for an intramural atrial lesion (4 mm depth). Although a 50 W ablation can create a 4 mm depth lesion with a short ablation time, it has a higher incidence of steam pops. However, the LI drop correlated with the temperature rise at depths of 2 mm, and a greater LI drop was highly related to the steam pops. Therefore, a 50 W ablation while monitoring the LI drop would be useful to safely reduce the procedure time of the PVI.

## 5 LIMITATIONS

There were several limitations to our study. Firstly, our experiment was performed in an ex‐vivo experimental model. Our results might represent an experimental error. Various conditions differed between the RF ablation in this experimental model and the real clinical RF ablation. The reaction of living tissue and the CF changes due to the heart beats, respirations, or anatomy, were not considered in our results. Although catheter orientation is related to the LI drop and lesion formation, the catheter contact in this study was performed with a perpendicular setting. Our results might differ from those with other catheter angles. Secondly, our experiment was performed in normal ventricular myocardium for a quantitative purpose, and those results might differ between atrial tissue and damaged scar tissue.

## 6 CONCLUSION

The lesion depth and diameter and LI drop with the StablePoint™ revealed non‐linear changes. The LI drop correlated with the lesion formation, and the ablation lesion was mainly created during the rapidly increasing phase. A greater lesion cannot be created with a longer ablation than the 90% decay time of the LI drop. An ablation shorter than the 90% decay time of the LI drop would be preferable for an effective ablation.

## AUTHOR CONTRIBUTIONS

HM, and RK, study conception and design; DK, NT, MT, WS, KT, and KM, data collection and data analysis; and HF, YI, TA, and SN manuscript revision.

## FUNDING INFORMATION

No funding was required for this study.

## CONFLICT OF INTEREST

Authors declare no conflict of interests for this article.

## CODE AVAILABILITY

Available upon request.

## Data Availability

Available upon request.

## References

[1] Calkins H , Hindricks G , Cappato R , Kim YH , Saad EB , Aguinaga L , et al. 2017 HRS/EHRA/ECAS/APHRS/SOLAECE expert consensus statement on catheter and surgical ablation of atrial fibrillation: executive summary. J Arrhythm. 2017;33(5):369–409. 29021841 10.1016/j.joa.2017.08.001 PMC5634725

[2] Haines DE . Determinants of lesion size during radiofrequency catheter ablation: the role of electrode‐tissue contact pressure and duration of energy delivery. J Cardiovasc Electrophysiol. 1991;2:509–15.

[3] Skrumeda LL , Mehra R . Comparison of standard and irrigated radiofrequency ablation in the canine ventricle. J Cardiovasc Electrophysiol. 1998;9(11):1196–205. 9835264 10.1111/j.1540-8167.1998.tb00092.x

[4] Kuck KH , Reddy VY , Schmidt B , Natale A , Neuzil P , Saoudi N , et al. A novel radiofrequency ablation catheter using contact force sensing: Toccata study. Heart Rhythm. 2012;9(1):18–23. 21872560 10.1016/j.hrthm.2011.08.021

[5] Reddy VY , Shah D , Kautzner J , Schmidt B , Saoudi N , Herrera C , et al. The relationship between contact force and clinical outcome during radiofrequency catheter ablation of atrial fibrillation in the TOCCATA study. Heart Rhythm. 2012;9(11):1789–95. 22820056 10.1016/j.hrthm.2012.07.016

[6] Weiss C , Antz M , Eick O , Eshagzaiy K , Meinertz T , Willems S . Radiofrequency catheter ablation using cooled electrodes: impact of irrigation flow rate and catheter contact pressure on lesion dimensions. Pacing Clin Electrophysiol. 2002;25(4 Pt 1):463–9. 11991372 10.1046/j.1460-9592.2002.00463.x

[7] Ruffy R , Imran MA , Santel DJ , Wharton JM . Radiofrequency delivery through a cooled catheter tip allows the creation of larger endomyocardial lesions in the ovine heart. J Cardiovasc Electrophysiol. 1995;6(12):1089–96. 8720209 10.1111/j.1540-8167.1995.tb00386.x

[8] Petersen HH , Chen X , Pietersen A , Svendsen JH , Haunsø S . Temperature‐controlled irrigated tip radiofrequency catheter ablation: comparison of in vivo and in vitro lesion dimensions for standard catheter and irrigated tip catheter with minimal infusion rate. J Cardiovasc Electrophysiol. 1998;9(4):409–14. 9581956 10.1111/j.1540-8167.1998.tb00928.x

[9] Matsuura G , Fukaya H , Ogawa E , Kawakami S , Mori H , Saito D , et al. Catheter contact angle influences local impedance drop during radiofrequency catheter ablation: insight from a porcine experimental study with 2 different LI‐sensing catheters. J Cardiovasc Electrophysiol. 2022;33(3):380–8. 35018687 10.1111/jce.15356

[10] Masnok K , Watanabe N . Catheter contact area strongly correlates with lesion area in radiofrequency cardiac ablation: an ex vivo porcine heart study. J Interv Card Electrophysiol. 2022;63(3):561–72. 34499311 10.1007/s10840-021-01054-3 PMC9151538

[11] Calzolari V , de Mattia L , Indiani S , Crosato M , Furlanetto A , Licciardello C , et al. In vitro validation of the lesion size index to predict lesion width and depth after irrigated radiofrequency ablation in a porcine model. JACC Clin Electrophysiol. 2017;3(10):1126–35. 29759495 10.1016/j.jacep.2017.08.016

[12] Das M , Loveday JJ , Wynn GJ , Gomes S , Saeed Y , Bonnett LJ , et al. Ablation index, a novel marker of ablation lesion quality: prediction of pulmonary vein reconnection at repeat electrophysiology study and regional differences in target values. Europace. 2017;19(5):775–83. 27247002 10.1093/europace/euw105

[13] Kanamori N , Kato T , Sakagami S , Saeki T , Kato C , Kawai K , et al. Optimal lesion size index to prevent conduction gap during pulmonary vein isolation. J Cardiovasc Electrophysiol. 2018;29(12):1616–23. 30176083 10.1111/jce.13727

[14] Taghji P , El Haddad M , Phlips T , Wolf M , Knecht S , Vandekerckhove Y , et al. Evaluation of a strategy aiming to enclose the pulmonary veins with contiguous and optimized radiofrequency lesions in paroxysmal atrial fibrillation: a pilot study. JACC Clin Electrophysiol. 2018;4(1):99–108. 29600792 10.1016/j.jacep.2017.06.023

[15] Mori H , Kato R , Sumitomo N , Ikeda Y , Goto K , Tanaka S , et al. Relationship between the ablation index, lesion formation, and incidence of steam pops. J Arrhythm. 2019;35(4):636–44. 31410234 10.1002/joa3.12195 PMC6686293

[16] Garrott K , Gams A , Laughner J , Lehn L , Gutbrod S , Hamann J . 1319Local impedance on a force sensing catheter predicts volumetric lesion temperature changes. EP Europace. 2020;22(Supplement_1):euaa162.248.

[17] Garrott K , Laughner J , Gutbrod S , Sugrue A , Shuros A , Sulkin M , et al. Combined local impedance and contact force for radiofrequency ablation assessment. Heart Rhythm. 2020;17(8):1371–80. 32240822 10.1016/j.hrthm.2020.03.016

[18] Kawano D , Mori H , Kato R , Tsutsui K , Ikeda Y , Sumitomo N , et al. The optimal ablation setting for a local impedance guided catheter in an in vitro experimental model. J Cardiovasc Electrophysiol. 2021;32(8):2069–76. 34185348 10.1111/jce.15136

[19] Tsutsui K , Kawano D , Mori H , Kato R , Ikeda Y , Sumitomo N , et al. Characteristics and optimal ablation settings of a novel, contact‐force sensing and local impedance‐enabled catheter in an ex vivo perfused swine ventricle model. J Cardiovasc Electrophysiol. 2021;32(12):3187–94. 34559441 10.1111/jce.15253

[20] Simmers TA , de Bakker JM , Wittkampf FH , Hauer RN . Effects of heating with radiofrequency power on myocardial impulse conduction: is radiofrequency ablation exclusively thermally mediated? J Cardiovasc Electrophysiol. 1996;7(3):243–7. 8867298 10.1111/j.1540-8167.1996.tb00521.x

[21] Yokoyama K , Nakagawa H , Shah DC , Lambert H , Leo G , Aeby N , et al. Novel contact force sensor incorporated in irrigated radiofrequency ablation catheter predicts lesion size and incidence of steam pop and thrombus. Circ Arrhythm Electrophysiol. 2008;1(5):354–62. 19808430 10.1161/CIRCEP.108.803650

[22] Barkagan M , Leshem E , Rottmann M , Sroubek J , Shapira‐Daniels A , Anter E . Expandable lattice electrode ablation catheter: a novel radiofrequency platform allowing high current at low density for rapid, titratable, and durable lesions. Circ Arrhythm Electrophysiol. 2019;12(4):e007090. 30943762 10.1161/CIRCEP.118.007090 PMC6652200

[23] Ho SY , Sanchez‐Quintana D , Cabrera JA , Anderson RH . Anatomy of the left atrium: implications for radiofrequency ablation of atrial fibrillation. J Cardiovasc Electrophysiol. 1999;10(11):1525–33. 10571372 10.1111/j.1540-8167.1999.tb00211.x

[24] Hall B , Jeevanantham V , Simon R , Filippone J , Vorobiof G , Daubert J . Variation in left atrial transmural wall thickness at sites commonly targeted for ablation of atrial fibrillation. J Interv Card Electrophysiol. 2006;17(2):127–32. 17226084 10.1007/s10840-006-9052-2

